# The R18 Polyarginine Peptide Is More Effective Than the TAT-NR2B9c (NA-1) Peptide When Administered 60 Minutes after Permanent Middle Cerebral Artery Occlusion in the Rat

**DOI:** 10.1155/2016/2372710

**Published:** 2016-05-10

**Authors:** D. Milani, N. W. Knuckey, R. S. Anderton, J. L. Cross, B. P. Meloni

**Affiliations:** ^1^School of Health Sciences, The University of Notre Dame Australia, Fremantle, WA 6160, Australia; ^2^Western Australian Neuroscience Research Institute, Nedlands, WA 6009, Australia; ^3^Department of Neurosurgery, Sir Charles Gairdner Hospital, QEII Medical Centre, Nedlands, WA 6009, Australia; ^4^Centre for Neuromuscular and Neurological Disorders, The University of Western Australia, Nedlands, WA 6009, Australia

## Abstract

We examined the dose responsiveness of polyarginine R18 (100, 300, and 1000 nmol/kg) when administered 60 minutes after permanent middle cerebral artery occlusion (MCAO). The TAT-NR2B9c peptide, which is known to be neuroprotective in rodent and nonhuman primate stroke models, served as a positive control. At 24 hours after MCAO, there was reduced total infarct volume in R18 treated animals at all doses, but this reduction only reached statistical significance at doses of 100 and 1000 nmol/kg. The TAT-NR2B9c peptide reduced infarct volume at doses of 300 and 1000 nmol/kg, but not to a statistically significant extent, while the 100 nmol/kg dose was ineffective. The reduction in infarct volume with R18 and TAT-NR2B9c peptide treatments was mirrored by improvements in one or more functional outcomes (namely, neurological score, adhesive tape removal, and rota-rod), but not to a statistically significant extent. These findings further confirm the neuroprotective properties of polyarginine peptides and for R18 extend its therapeutic time window and dose range, as well as demonstrating its greater efficacy compared to TAT-NR2B9c in a severe stroke model. The superior neuroprotective efficacy of R18 over TAT-NR2B9c highlights the potential of this polyarginine peptide as a lead candidate for studies in human stroke.

## 1. Introduction

While the incidence of stroke is falling in developed countries, it remains a leading cause of death and disability worldwide, with an increasing global disease burden due to an aging population, as well as the ongoing epidemics of diabetes, hypertension, and obesity [1]. In terms of acute therapies, for ischaemic stroke, reperfusion therapy using tPA (tissue plasminogen activator) alone or more recently in combination with thrombectomy is by far the most effective treatment intervention currently available [2–6]. However, despite the success of tPA/thrombectomy therapy, the number of stroke patients that receive this treatment is relatively small. This is due to a combination of factors including the narrow therapeutic time window for tPA/thrombectomy (3–4.5 h after stroke), delays in patients obtaining medical care, the requirement for a brain scan to exclude haemorragic stroke, and the need for highly trained personnel and specialised equipment to perform the intervention. Given these limitations, the search continues for a neuroprotective agent that can be safely administered early after stroke onset to limit the extent of brain injury after stroke and that can be used when reperfusion interventions cannot be implemented. Additionally, any neuroprotective treatment that improves the efficacy, safety, and therapeutic window for tPA/thrombectomy would be of great clinical significance.

In terms of neuroprotective agents, our laboratory has recently demonstrated that polyarginine and arginine-rich peptides have potent neuroprotective properties in* in vitro* injury models that mimic the effects of stroke [7–9]. Moreover, we have extended these* in vitro* findings by demonstrating that the polyarginine peptides R9, R12, and R18 significantly reduce infarct volume in a permanent middle cerebral artery occlusion (MCAO) stroke model [8, 10]. Based on these* in vitro* and* in vivo* findings, we have recently proposed [8, 9] that arginine-rich peptides including “neuroprotective peptides” fused to arginine-rich cell penetrating peptides (e.g., TAT-NR2B9c [11] and TAT-JNKI-1 [12]) represent a new class of neuroprotective agents for which arginine residues are critical for neuroprotection.

In the present study, we further evaluate the efficacy of the R18 polyarginine peptide by examining its dose responsiveness and by extending the treatment administration time from 30 minutes to 60 minutes after permanent MCAO. In parallel, the study compares the efficacy of R18 with that of TAT fused NR2B9c peptide (TAT-NR2B9c), which has previously been demonstrated to be neuroprotective in various rodent and nonhuman primate stroke models and to reduce ischaemic brain lesions in humans following endovascular repair of ruptured aneurysms [11, 13–15]. 

## 2. Materials and Methods 

### 2.1. Peptides Used in the Study

The R18 (H-RRRRRRRRRRRRRRRRRR-OH) and TAT-NR2B9c (H-YRKKRRQRRR-KLSSIESDV-OH, also known as NA-1) peptides used in the study were synthesised by Mimotopes (Melbourne, Australia). The peptides were HPLC-purified to 98% purity and were subject to peptide hydrolysis and amino acid liquid chromatography analysis to obtain a precise measurement of peptide content (Mimotopes). The peptides were prepared in 0.9% sodium chloride for injection (Pfizer, Perth, Australia), aliquoted into a 650 *μ*L volume in a 3 mL syringe and stored at −20°C until use.

### 2.2. Surgical Procedure for Permanent Middle Cerebral Artery Occlusion

The surgical procedures for permanent middle cerebral artery occlusion (MCAO) as well as behavioural and histologic assessment were performed in accordance with the Animal Ethics Committee of the University of Western Australia and following the guidelines outlined by the* Australian Code for the Care and Use of Animals for Scientific Purposes*.

The filament permanent MCAO stroke model as performed in our laboratory has been described previously [10, 16]. Briefly, male Sprague-Dawley rats weighing 275–340 g that had been fasted overnight underwent facemask anesthesia with 4% of isoflurane (mix 30% oxygen/70% nitrous oxide) and maintenance with 2% isoflurane. The tail artery was cannulated to allow blood pressure monitoring and for measurement of arterial blood gases (pO_2_, pCO_2_), pH, and glucose. The MCAO procedure was considered successful if there was a >25% decrease from baseline in cerebral blood flow after insertion of the filament, as measured by laser Doppler flowmetry. During surgery, body temperature was closely monitored using a rectal probe (Physitemp Instruments, Clifton, USA) and maintained at 37–37.8°C, with fan heating or cooling, as required.

At sixty minutes after MCAO rats were treated intravenously through the right internal jugular vein using an infusion pump with the vehicle (0.9% sodium chloride, 600 *μ*L over 6 min) or with three different doses of the peptide (R18 or TAT-NR2B9c: 100, 300, or 1000 nmol/kg, 600 *μ*L over 6 min). Treatments were randomised and all procedures were performed while being blinded to treatments.

Fifty male Sprague-Dawley rats underwent surgery for permanent MCAO. Eight animals were excluded from the study: five animals were excluded due to an insufficient decrease in cerebral blood flow following MCAO, one was excluded due to death during anesthetic induction, and two animals were excluded because no obvious infarct lesion was detected 24 hours after MCAO (one saline and one R18 100 nmol/kg treated animal). A further six animals died several hours before the 24-hour post-MCAO study end-point but were still included in the final infarct volume analysis. These animals comprised two R18 treated (one 100 nmol/kg and one 1000 nmol/kg) and four TAT-NR2B9c treated (two 100 nmol/kg, one 300 nmol/kg, and one 1000 nmol/kg) animals. While the exact cause of the deaths could not be determined, it is possible that it reflects the severity of the stroke in this model, which is known to result in up to 90% of the affected hemisphere being infarcted by the stroke. For infarct volume analysis each treatment group consisted of six animals. Due to animal deaths before the 24-hour study end-point, four to six animals per group were available for behavioural testing.

### 2.3. Postsurgical Monitoring

The body temperature of animals was measured every 30–60 minutes using a rectal probe for at least 2 hours after surgery and maintained between 37.0 and 37.8°C. To avoid hypothermia, rat cages were placed on a heating mat during the postsurgical monitoring and housed in a holding room maintained at 26–28°C. If necessary, additional heating or cooling was performed by applying fan heating or a cold water spray.

### 2.4. Infarct Volume Assessment

Infarct volume was assessed 24 hours after MCAO as previously described [17]. Briefly 2 mm cerebral coronal brain slices were stained in 3% 2,3,5-triphenyltetrazolium chloride (Sigma-Aldrich, St. Louis, USA). Digital images of coronal brain slices were acquired and analysed using ImageJ software (3rd edition, NIH, Bethesda, USA) by an operator blind to treatment status. The total infarct volume was determined by measuring the areas of infarcted tissue on both sides of the 2 mm sections and corrected for cerebral oedema [17].

### 2.5. Behavioural Testing

In order to assess if any treatment improved sensorimotor outcomes, three behavioural tests were performed 24 hours after MCAO. A neurological assessment was performed using a five-point scale (0−5) developed by Bederson et al. [18]. Scores range between 0 for no deficits, 1 for flexed forepaw, 2 for inability to resist lateral push, 3 for circling, 4 for agitated circling, and 5 for unresponsiveness to stimulation/stupor. The adhesive tape removal test is a bilateral asymmetry paw test to assess sensorimotor impairment [19]. Adhesive tape (Diversified Biotech, Dedham, USA) 10 mm × 10 mm in size was placed on the palmar surface of the forepaw and the time taken for the first attempt to remove tape (time to detect tape), the time taken to remove the tape, and the number of attempts to remove tape were recorded. Each forelimb was assessed sequentially starting with the unaffected side (right side) with animals having a maximum of 120 seconds to complete the task (normal rats usually take between 5 and 30 sec to remove the tape). Each rat was assessed three times on the day prior to the surgery and once 24 hours after MCAO. The rota-rod test assesses balance and coordination by assessing a rat's ability to keep walking on a rotating rod, with the speed of rotation being progressively increased from 4 to 40 revolutions per minute. The time the animal falls was recorded.

### 2.6. Statistical Analysis

Total infarct volume and physiological parameters were evaluated by analysis of variance (ANOVA) followed by Fisher's* post hoc* analysis. The neurological assessment measurements were analysed using Kruskal-Wallis test. Data from adhesive tape removal and rota-rod tests were analysed using ANOVA followed by Scheffé's multiple comparison* post hoc* analysis. *P* < 0.05 was considered as significant. Data are presented as mean ± standard deviation (SD).

## 3. Results 

### 3.1. Physiological Data, Infarct Volume Measurements, and Animal Deaths

Physiological parameters measured during surgery and before MCAO were within the normal range and did not differ significantly between animal treatment groups (Table 1).

**Figure 1:**
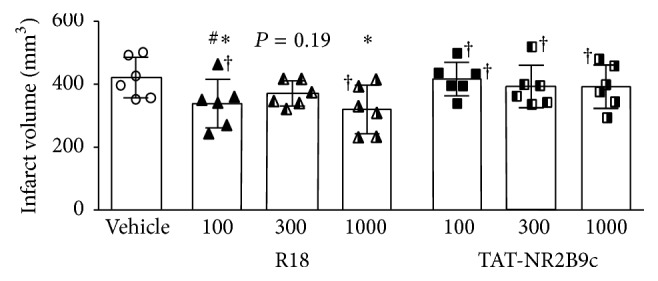
Infarct volume measurements 24 hours after permanent MCAO. Treatments were administered intravenously (saline vehicle or R18 and TAT-NR2B9c peptide at 100, 300, or 1000 nmol/kg; 600 *μ*L volume over 6 min) 60 minutes after MCAO. Values are mean ± SD. ^*∗*^ *P* < 0.05 when compared to the vehicle control group and ^#^ *P* < 0.05 when compared to the TAT-NR2B9c 100 nmol/kg group. † denotes animals that died several hours before the 24-hour post-MCAO study end-point but were still included in the final infarct volume analysis.

Data on the mean total infarct volumes for each treatment group are presented in Figure 1. These results show that the R18 peptide significantly reduced infarct volume at doses of 100 nmol/kg and 1000 nmol/kg by 19.7% (*P* = 0.043) and 24% (*P* = 0.013), respectively, while, at the 300 nmol/kg, infarct volume was reduced by 12% (*P* = 0.19), albeit not to a statistically significant extent. By contrast, while the TAT-NR2B9c peptide at doses of 300 nmol/kg and 1000 nmol/kg reduced infarct volume by 6.8% (*P* = 0.56) and 7% (*P* = 0.55), respectively, these effects were not statistically significant. At 100 nmol/kg, TAT-NR2B9c was ineffective in reducing infarct volume. In comparative terms, at 100 nmol/kg R18 was significantly more effective in reducing infarct volume than TAT-NR2B9c (19.7% versus 1.1%, *P* = 0.045).

### 3.2. Functional Outcome Assessment

**Figure 2:**
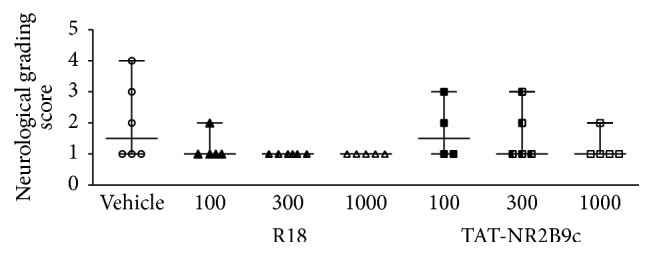
Neurological grading scores 24 hours after permanent MCAO (0 = no deficit, 5 = major deficit) for saline (vehicle) and peptide (R18 and TAT-NR2B9c at 100, 300, or 1000 nmol/kg) treatment groups. Lines on graph indicate range and median for neurological scores.

**Figure 3 fig3:**
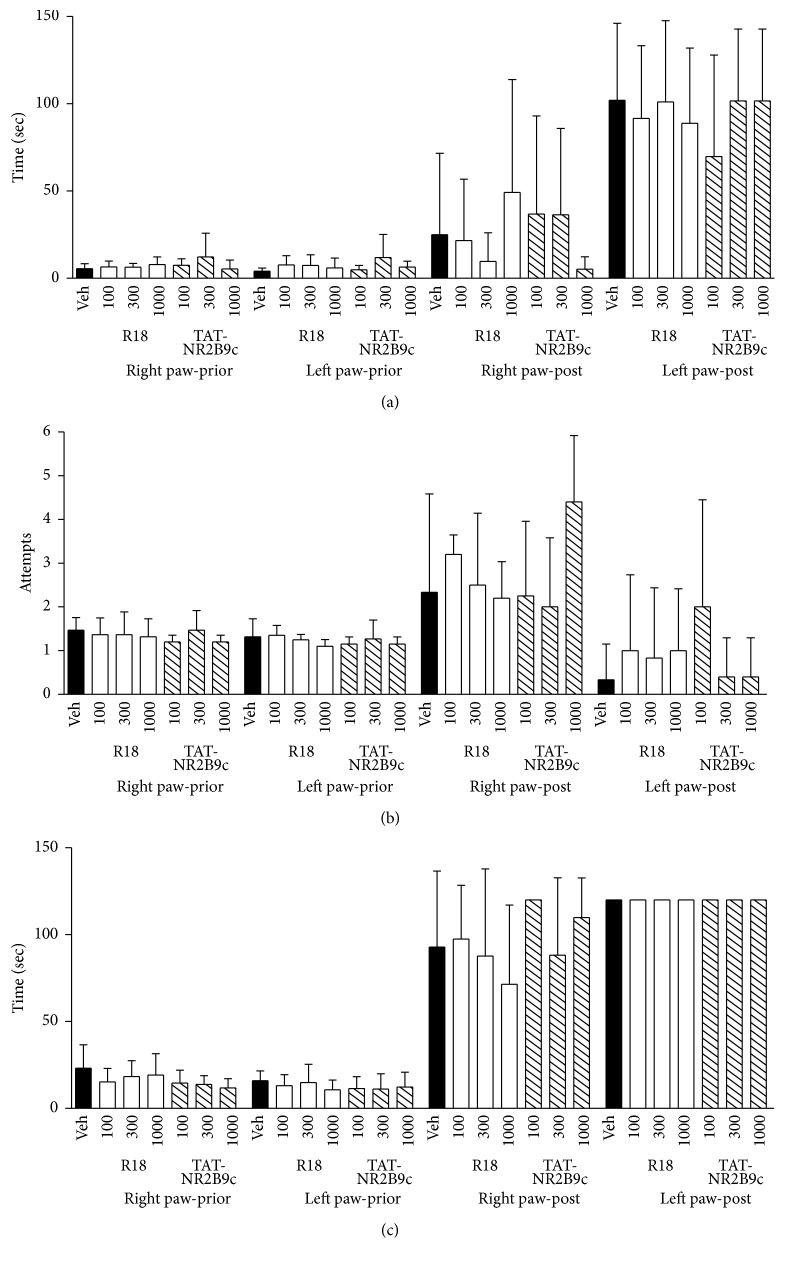
Functional assessment measurements using adhesive tape removal test before and 24 hours after MCAO for saline (vehicle) and peptide (R18 and TAT-NR2B9c at 100, 300, or 1000 nmol/kg) treatment groups. (a) Time to detect tape. (b) Number of attempts to remove tape. (c) Time to remove tape. Values are mean ± SD. Maximum time allowed for adhesive tape removal was 120 seconds.

**Figure 4:**
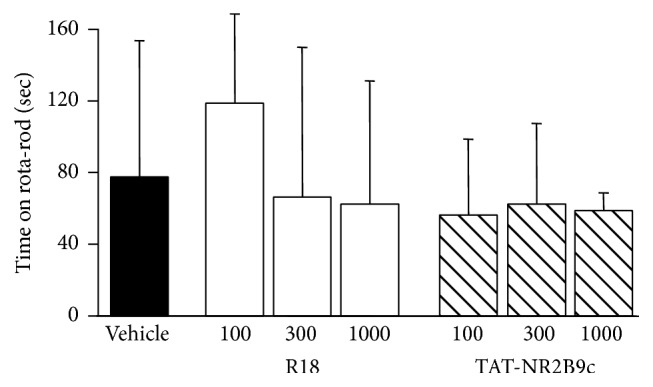
Rota-rod performance 24 hours after permanent MCAO for saline (vehicle) and peptide (R18 and TAT-NR2B9c at 100, 300, or 1000 nmol/kg) treatment groups. Values are mean ± SD.

Although not statistically significant, there was a trend towards improvement in the performance in some of the behavioural parameters measured for R18 and TAT-NR2B9c treatment groups (Figures 2 [Fig fig3]–4). Neurological scores for 100, 300, and 1000 nmol/kg R18 treatment animals showed improved outcomes compared with the vehicle-treated controls (Figure 2). By contrast, for TAT-NR2B9c, only the 1000 nmol/kg treatment group was associated with an improvement in neurological score compared to vehicle. Measurements for the adhesive tape test after MCAO were variable; however, treatment with the 300 nmol/kg R18 or 1000 nmol/kg TAT-NR2B9c appeared to improve the time required to detect tape from the right paw of the nonaffected forelimb, while the 100 nmol/kg TAT-NR2B9c treatment appeared to improve the time required to detect tape from the left paw (Figure 3(a)). Similarly, the number of attempts required to remove the tape from the right and left paw was increased in animals treated with 1000 nmol/kg and 100 nmol/kg TAT-NR2B9c, respectively (Figure 3(b)). Additionally, treatment with 1000 nmol/kg R18 was associated with the shortest time to remove tape from the right paw (Figure 3(c)). For the rota-rod test, the group receiving 100 nmol/kg R18 was the only treatment group that displayed an increased time to remain on the rotating cylinder when compared to vehicle (118 sec versus 77 sec, Figure 4).

### 3.3. Weight Loss Measurement

**Figure 5:**
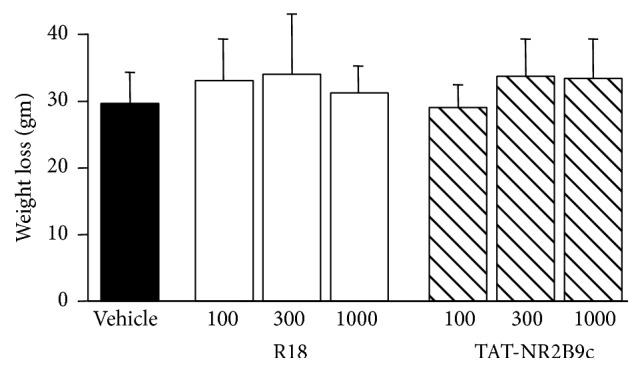
Weight loss at 24 hours after permanent MCAO for saline (vehicle) and peptide (R18 and TAT-NR2B9c at 100, 300, or 1000 nmol/kg) treatment groups. Values are mean ± SD.

All groups recorded a loss in body weight 24 hours after MCAO ranging from 28.5 grams for the TAT-NR2B9c 100 nmol/kg treatment group to ≈34.5 g for the 300 nmol/kg R18 and TAT-NR2B9c treatment groups (Figure 5).

## 4. Discussion

The results of the present study add to our previous findings, which showed that 1000 nmol/kg R18 when administered 30 minutes after permanent MCAO significantly reduces infarct volume in the rat [10]. Importantly, we now show that R18 is effective over an even wider therapeutic window (60 min) and broader dose range (100–1000 nmol/kg) and that, on balance, R18 is more effective than the extensively characterised neuroprotective peptide, TAT-NR2B9c. Treatment with R18, as well as to a lesser extent TAT-NR2B9c, resulted in some functional recovery as assessed by behavioural tests, but not to statistically significant levels, which most likely reflects the severity of the stroke model used coupled with the relatively small numbers of animals in the study. Notwithstanding these limitations, our findings highlight the potential clinical applicability of R18 as a therapeutic intervention in stroke, especially in light of evidence that it is superior as a neuroprotective agent to TAT-NR2B9c, which is planned to enter a phase 3 clinical trial in stroke patients [20]. The superior neuroprotective efficacy of R18 compared to TAT-NR2B9c is consistent with our* in vitro* findings in a glutamic acid induced neuronal excitotoxicity model of cell death [8].

The TAT-NR2B9c peptide has been shown to be neuroprotective in rodent [11, 21–25] and nonhuman primate stroke models [13, 14] and has been found to be safe and cause a nonsignificant reduction in ischaemic brain lesions in patients undergoing aneurysm surgery [15]. The NR2B9c peptide (KLSSIESDV) is derived from the intracellular terminal carboxyl region of the N-methyl-D-aspartate (NMDA) receptor NR2B subunit protein [11] and is fused to the arginine-rich TAT peptide (YRKKRRQRRR) to allow entry into the brain and neuronal cells. The NR2B9c peptide was designed to act as a competitive inhibitor of the PSD-95 adaptor protein (postsynaptic density-95) binding to the NR2B subunit protein and, in doing so, to block downstream cell signaling associated with overstimulation of the NMDA receptor, leading to nitric oxide synthase activation and subsequent production of nitric oxide; however, we [9] and others [25, 26] have proposed other mechanisms for neuroprotection.

As an alternative mechanism, we have proposed that the neuroprotective properties of TAT-NR2B9c are largely mediated by the TAT peptide itself [9], which we [7, 27] and others [28, 29] have previously reported to display modest neuroprotective properties. Furthermore, due to the TAT peptide's arginine content and positive charge, it is likely to possess a similar mode of action as polyarginine and arginine-rich peptides [8, 9]. For arginine-rich peptides, we have previously hypothesised that at least in part neuroprotection is related to the ability of these peptides to transverse cell membranes and, in doing so, decrease the levels of cell surface ion channels and receptors, thereby reducing the toxic influx of calcium that occurs in neurons following cerebral ischaemia [8, 9]. This mechanism of action is in line with the confirmed ability of arginine-rich peptides to reduce glutamate excitotoxic calcium influx in cortical neurons [8, 9, 30–32] and evoke receptor currents in NR1-NR2 NMDA receptor-expressing oocytes [33], as well as the observation that peptide neuroprotective efficacy correlates with peptide endocytic or cell membrane transversing properties [34]. In addition, several studies have demonstrated that TAT fused peptides and arginine-rich cell penetrating peptides can reduce the expression of cell surface ion channels and receptors in neurons [26, 31, 35–38] and other cells [39].

There is evidence to indicate that arginine residues are critical elements for peptide and protein mitochondrial uptake [40–43] and that arginine-rich peptides exert beneficial effects on mitochondria. For example, in isolated rat liver mitochondria, cationic tetra- and polycationic peptides and especially those containing arginine were highly effective in blocking calcium induced mitochondrial swelling and in maintaining membrane potential [44]. Similarly, cationic compounds including tetrapeptides containing an arginine residue (e.g., SS-20, SS-31) or biguanidines (e.g., metformin) have been shown to target mitochondria and exert positive effects on the organelle by limiting complex I activity and reactive oxygen species production [41, 45], inhibiting the opening of the mitochondrial permeability transition pore [46], protecting cristae architecture [47], accelerating ATP recovery [47], and preventing cytochrome c release [48]. While the exact mechanisms for these beneficial effects on mitochondria are not fully known, the ability of cationic guanidino groups to interact with anionic phosphate groups of mitochondrial membrane phospholipids especially the inner membrane phospholipid cardiolipin (−2 net charge) may be a contributing factor.

Recently, Marshall et al. [49] confirmed the neuroprotective properties of polyarginine peptides in an* in vivo* NMDA-induced retinal ganglion cell excitotoxicity model and provided evidence that the peptides reduce neuronal mitochondrial oxidative stress. Furthermore, it was demonstrated in HEK293 cells that polyarginine peptides localise to mitochondria and reduce mitochondrial respiration, membrane potential, and levels of reactive oxygen species [49]. It is also interesting to note that Marshall et al. [49] found that polyarginine peptides taken up by retinal ganglion cells are localised within small spherical cytoplasmic structures, which the authors suggested to be mitochondria. Also of interest are recent studies surrounding the arginine-rich Borna disease viral mitochondrial-targeting protein, X. The full-length protein X and X-derived peptide fused to a cell penetrating peptide display neuronal, axonal, and mitochondrial protective properties [50, 51].

Another mechanism whereby arginine-rich peptides exert a neuroprotective effect may be related to the ability of polyarginine and arginine-rich peptides to inhibit the proteolytic activity of proprotein convertases (e.g., furin and PC4 [52, 53]), cathepsin C [54], and the proteasome [55, 56], an effect that may be beneficial following brain ischaemia. For example, furin is ubiquitously expressed calcium-dependent convertase responsible for the activation of membrane bound proteins, including metalloproteinases, which are known to have adverse effects on the blood brain barrier following stroke [57]. Similarly, treatments known to inhibit the proteasome which is responsible for the degradation of short-lived cytosolic proteins are known to reduce brain injury in stroke [58, 59].

Taken together, the results from the current study support other findings from our laboratory [8–10] and suggest that polyarginine and arginine-rich peptides may represent a new class of neuroprotective agents with enormous clinical potential for the treatment of acute and chronic neurological injuries. Importantly, it is possible that the reported beneficial effects of arginine-rich cell penetrating peptides fused to a “neuroprotective peptide” in animal studies of acute brain injury are largely attributable to the effects of the arginine residues contained within the peptide [9]. This adds to the growing weight of evidence suggesting that arginine-rich peptides (including R18) may be beneficial in a range of acute clinical neurological disorders. There is also evidence that arginine-rich peptides may improve functional recovery from central nervous system injury as evidenced by experimental studies on the effects of TAT-NR2B9c in stroke [60] and TAT-ISP in spinal cord injury [61]. Consequently, there is a growing body of evidence that supports the need for clinical studies on the effects of arginine-rich peptides to establish whether these peptides are equally beneficial in patients with stroke or other acute and chronic neurological disorders.

## Figures and Tables

**Table 1:** Physiological parameters (mean ± SD).

Parameter	Experimental groups						
	Vehicle (saline)	R18 (nmol/kg)			TAT-NR2B9c (nmol/kg)		
		100	300	1000	100	300	1000
PaO_2_, before MCAO	117.19 ± 22.44	114.66 ± 11.34	115.16 ± 13.79	124.16 ± 14.35	109.33 ± 9.04	117.83 ± 16.55	112.83 ± 12.93
PaCO_2_, before MCAO	38.33 ± 2.94	39 ± 3.57	39.5 ± 3.50	42.68 ± 6.17	43.66 ± 8.93	39.5 ± 5.78	39.83 ± 3.74
pH, before MCAO	7.36 ± 0.05	7.42 ± 0.09	7.37 ± 0.04	7.44 ± 0.06	7.38 ± 0.04	7.45 ± 0.04	7.39 ± 0.06
Glucose (mmol/L), before MCAO	8.0 ± 1.37	8.15 ± 0.68	7.81 ± 0.96	8.11 ± 1.00	7.21 ± 1.31	7.71 ± 0.81	7.50 ± 1.73
BP (mmHg), average during surgery	87.33 ± 4.17	88.33 ± 7.06	84 ± 5.13	91.6 ± 5.44	91 ± 3.09	83.66 ± 6.34	88 ± 3.68
Temperature (°C), average 2 h after surgery	37.51 ± 0.16	37.37 ± 0.25	37.46 ± 0.29	37.46 ± 0.21	37.43 ± 0.25	37.53 ± 0.17	37.52 ± 0.21
